# Periostin in Allergy and Inflammation

**DOI:** 10.3389/fimmu.2021.722170

**Published:** 2021-08-27

**Authors:** Eva Sonnenberg-Riethmacher, Michaela Miehe, Dieter Riethmacher

**Affiliations:** ^1^Department of Biomedical Sciences, School of Medicine, Nazarbayev University, Nur-Sultan, Kazakhstan; ^2^Department of Human Development and Health, School of Medicine, University of Southampton, Southampton, United Kingdom; ^3^Department of Biological and Chemical Engineering – Immunological Biotechnology, Aarhus University, Aarhus, Denmark

**Keywords:** periostin, isoforms, allergy, chronic inflammation, IBD, diagnostic marker

## Abstract

Matricellular proteins are involved in the crosstalk between cells and their environment and thus play an important role in allergic and inflammatory reactions. Periostin, a matricellular protein, has several documented and multi-faceted roles in health and disease. It is differentially expressed, usually upregulated, in allergic conditions, a variety of inflammatory diseases as well as in cancer and contributes to the development and progression of these diseases. Periostin has also been shown to influence tissue remodelling, fibrosis, regeneration and repair. In allergic reactions periostin is involved in type 2 immunity and can be induced by IL-4 and IL-13 in bronchial cells. A variety of different allergic diseases, among them bronchial asthma and atopic dermatitis (AD), have been shown to be connected to periostin expression. Periostin is commonly expressed in fibroblasts and acts on epithelial cells as well as fibroblasts involving integrin and NF-κB signalling. Also direct signalling between periostin and immune cells has been reported. The deposition of periostin in inflamed, often fibrotic, tissues is further fuelling the inflammatory process. There is increasing evidence that periostin is also expressed by epithelial cells in several of the above-mentioned conditions as well as in cancer. Augmented periostin expression has also been associated with chronic inflammation such as in inflammatory bowel disease (IBD). Periostin can be expressed in a variety of different isoforms, whose functions have not been elucidated yet. This review will discuss potential functions of periostin and its different isoforms in allergy and inflammation.

## Introduction

**Figure 1:**
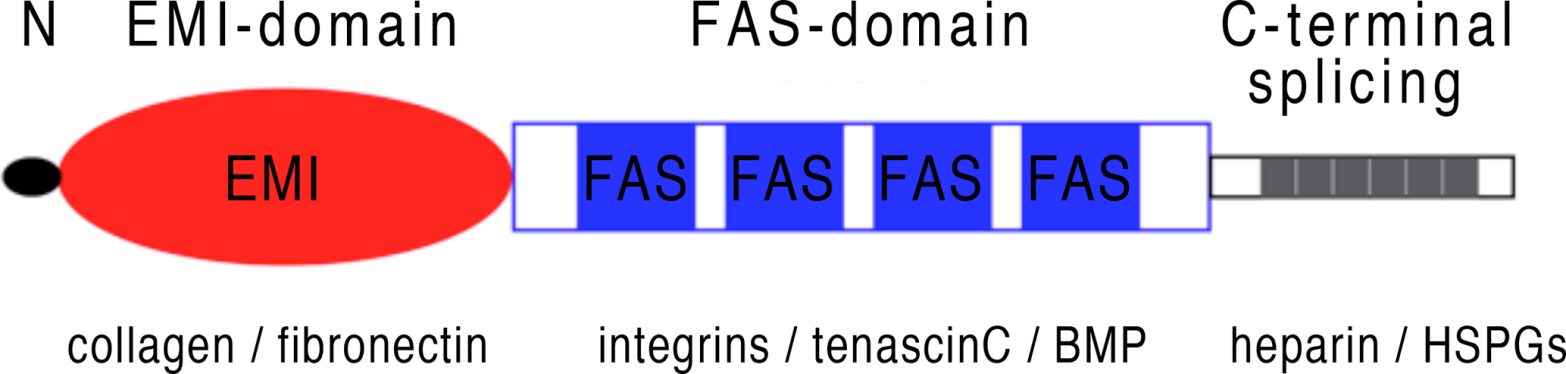
Periostin schematic structure: Periostin is a secretory protein with a multi-domain structure, consisting of a secretion signal peptide, a N-terminal cysteine-rich EMI domain, four internal FAS domains and a C-terminal hydrophilic domain that can be alternatively spliced. The EMI domain interacts with collagen and fibronectin, while the FAS domains can bind to integrins, tenascin-C and BMP. The C-terminal domain shows a high degree of alternative splicing, see also **Figure 3** and can bind to heparin and HSPGs.

**Figure 2:**
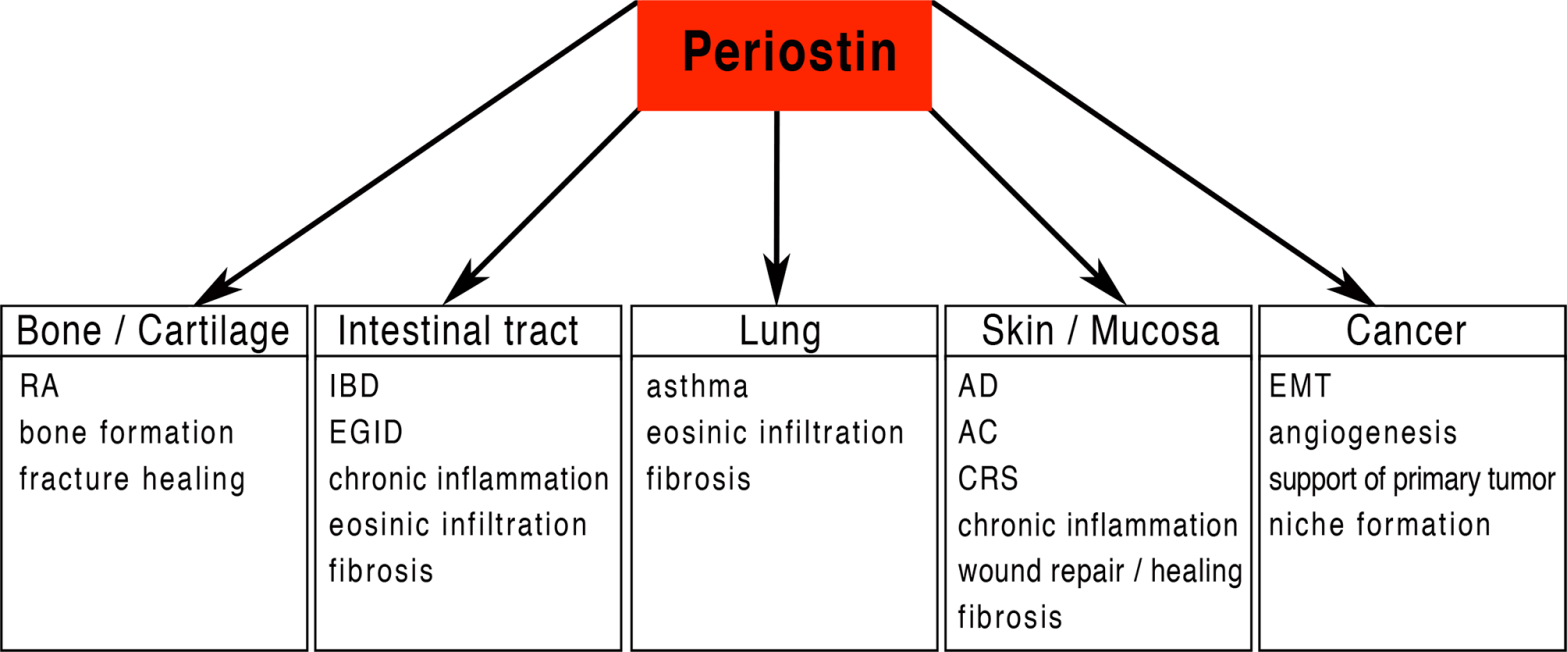
Periostin potential functional implications: Periostin is implicated in a large number of physiological and pathological processes including the attraction and infiltration of inflammatory cells in a variety of different organs/tissues. Periostin is in a key position for driving chronic inflammation and fibrosis in various tissues. RA, Rheumatoid arthritis; IBD, Inflammatory bowel disease; EGID, Eosinophilic gastrointestinal disorder; AD, Atopic dermatitis; AC, Allergic conjunctivitis; CRS, Chronic Rhinosinusitis; EMT, epithelial-mesenchymal transition.

Periostin is a matricellular protein originally isolated from osteoblasts and found to be preferentially expressed in the periosteum (1, 2). Periostin contains an N-terminal secretory signal peptide, followed by a cysteine-rich domain (EMI domain), four internal homologous repeats (FAS domains), and a C-terminal hydrophilic domain that is alternatively spliced (1–3) (**Figure 1**). The four internal repeats exhibit homology to the axon guidance protein fasciclin I that is involved in the development of the nervous system in invertebrates and therefore were named fasciclin domains. The EMI domain, named after a domain first described in the EMILIN family, can bind to collagen I and fibronectin, while the FAS-domains bind to tenascin-C and bone morphogenic protein 1 (BMP1) (4–6) (**Figure 1**). The C-terminal domain shows a high degree of alternative splicing, is known to bind heparin and heparan sulfate proteoglycans (HSPGs) and to modulate the binding mediated by the other domains (7). As a matricellular protein, periostin is mainly localised in the extracellular matrix (ECM) and some of its activities are mediated by its binding to cell surface receptors of the integrin family (8). Periostin has been shown to be differentially expressed (usually upregulated) in a variety of allergic manifestations, inflammation and tumours. Its expression in a tissue-specific context is regulated by several proteins, including TGF-β1 (1), bone morphogenetic proteins (BMP) 2 and 4 (9), various interleukins (IL-3, 4, 6 and 13) (10), erbB3 activation involving neuregulins (NRGs) (11), vascular endothelial growth factor, vitamin K and others (12). Periostin can influence other signalling pathways including the NF-κB, focal adhesion kinase (FAK), phosphatidylinositol 3-kinase (PI3K)/AKT and Yap/TAZ pathways through its interaction with α_v_-integrins, transmembrane receptors facilitating adhesion of cells to the ECM. It also regulates the expression of several other genes within its functionally connected network, including collagen, α-smooth muscle actin (α-SMA), TGF-β1 and various chemokines (Ccl2, Ccl4, Ccl5, Ccl7, Cxcl1, Cxcl2) (12–15). Different splice variants generate different isoforms of periostin (3), and it is conceivable to assume that the different isoforms could be contributing to the huge variety of functions reported for periostin. Among these multiple functions, listed in **Figure 2** and **Table 1**, there are epithelial-mesenchymal transition, niche formation, regeneration, tissue repair, inflammatory processes, fibrosis and angiogenesis (10, 35, 65–68). Regeneration and repair is delayed in the absence of periostin (60, 61, 69), however, its expression is not always beneficial as periostin can also be a driver of fibrosis (46, 70, 71). EMT has been shown to be involved in fibrosis (72) and periostin is an important player in EMT (73–75). Cell proliferation has been shown to be enhanced by periostin during normal development and in disease (70, 76–78). Angiogenesis, which is a prerequisite for a variety of processes in development and disease, is also positively influenced by the expression of periostin (66, 79). Another function of periostin is the facilitation of migration (11, 77, 80, 81). Several pathways activated by periostin during these processes have been elucidated (2, 4, 11, 35, 66, 73, 79, 80, 82). However, little information is available on the different isoforms and how they might contribute to these processes.

**Table 1:** Periostin as a diagnostic marker and/or potential therapeutic target in different organs, illnesses and processes.

Organ	Illness/Process	Periostin as diagnostic marker	Periostin as potential therapeutic target	References
**Bone/cartilage**	RA	Yes	?	(16–20)
	AS	Yes	?	(21)
	Bone formation	Yes	?	(21–23)
	Fracture healing	Reduced serum levels 48h after injury	Yes - periostin ↑	(24–27)
**Digestive tract**	IBD	No for children / ? for adults	Yes - periostin ↓	(28–30)
	EGID / EoE	No	?	(31–34)
**Lung**	Asthma	Yes	Yes - periostin ↓	(35–42)
	Eosinophilic infiltration	Yes	Yes - periostin ↓	(43–45)
	Fibrosis*	Yes	Yes - periostin ↓	(10, 41, 46–48,)
**Skin/mucosa**	AD	Yes	Yes - periostin ↓	(36, 37, 49–51)
	AC	Yes	Yes - periostin ↓	(52–54)
	CRS	Yes	Yes - periostin ↓	(55–59)
	Wound repair/healing	?	Yes - periostin ↑	(60) (heart) (61–63),
	Urticaria	?	?	(64)

In this table we listed several organs, illnesses and processes in which evidence suggests periostin fulfils important functions and could be valuable diagnostic marker or therapeutic target. For diagnostic marker a yes means that there is published literature that serum levels of periostin are altered in correlation to the disease status and or healthy controls in humans. For therapeutic target the question mark (?) expresses that currently there is no strong evidence that periostin is a potential candidate, while yes means that either increasing (↑) or decreasing (↓) periostin has shown beneficial effects in cell culture and/or animal experiments. The asterisk (*) next to fibrosis reflects that it is listed under lung but as a process applies to other organs as well (e.g. skin, heart, intestine). When searching https://clinicaltrials.gov/ with periostin there are >50 trials indicated with at least 10 listing periostin in the title or as intervention, demonstrating that there is increasing interest in periostin as a diagnostic marker or therapeutic agent. RA, Rheumatoid arthritis; AS, Ankylosing Spondylitis; IBD, Inflammatory bowel disease; EGID, Eosinophilic gastrointestinal disorder; EoE, eosinophilic esophagitis; AD, Atopic dermatitis; AC, Allergic conjunctivitis; CRS, Chronic Rhinosinusitis.Additional supporting references (24–27, 34, 42, 51, 62, 63).

The involvement of periostin has been well described across a spectrum of allergic, inflammatory and fibrotic conditions including inflammatory conditions of the respiratory tract (asthma, chronic obstructive pulmonary disease, allergic rhinitis, idiopathic pulmonary fibrosis), systemic sclerosis and scleroderma, atherosclerosis, fibrosis, renal interstitial fibrosis, hepatic fibrosis, inflammatory bowel disease and others (83). In this review, we will discuss the function of periostin in allergy and inflammation, specifically taking into account the isoforms identified so far.

## Periostin in Allergy and Chronic Inflammation

Periostin has been linked to allergy and inflammation in a variety of different organs (**Figure 2** and **Table 1**). It is widely accepted that type 2 (T2) immunity is dominant in allergic inflammation. Type 2 immunity cytokines interleukins IL-4, IL-5 and IL-13 are important in humoral immunity and protection from helminth infection and are central to the pathogenesis of many allergic inflammatory diseases (84, 85). IL-4, IL-5 and IL-13 are produced by T helper 2 (Th2) cells, follicular helper T cells, eosinophils, mast cells and basophils (86–89).

Binding of IL-4 and IL-13 to their receptors upregulates expression of periostin in a STAT-6 and SOX11 dependent manner, either directly or indirectly (36, 89–92). Furthermore, it was reported that periostin supports α_M_β_3_ integrin-mediated adhesion and migration of IL-5 stimulated eosinophils (43–45).

Periostin has been shown to be upregulated in the subepithelial region in asthma (10), AD (37, 49), allergic conjunctivitis (AC) (52), eosinophilic esophagitis (EoE) (31) and familial idiopathic pulmonary fibrosis (IPF) (47, 48). Periostin is also upregulated in chronic inflammations such as IBD and rheumatoid arthritis (RA) (16, 28, 29). Interestingly, IL-24 and STAT-3 as downstream effectors of periostin are enhancing the epithelial-barrier dysfunction thereby further fuelling the inflammatory process (92). All these illnesses entail inflammation followed by regeneration of tissue, in which locally expressed periostin plays an important part.

Macrophages are known to be involved in allergic diseases, however their specific roles are not fully understood. It is known that some macrophage types may exacerbate (M1, M2a and M2b) or reduce (M2c, M2d) allergic reactions (93). In asthma, lung-resident alveolar macrophages serve to maintain homeostasis in this organ by supressing inflammation, while macrophages derived from immigrating cells promote allergic reactions (94–96). Noteworthily, in an investigation on peripheral nerves, periostin was shown to chemotactically attract macrophages to the site of allergic reactions (97). However, Allard et al. did not further characterize which macrophage subcategories were attracted *via* periostin and which types of allergic reactions were enhanced or reduced. In that experimental setting however, absence of periostin leads to a lower macrophage infiltration, thereby lowering inflammation and slowing down disease progression. This suggests that periostin attracts mainly pro-inflammatory macrophages and may apply to other organs and inflammatory situations as well.

## Asthma

Periostin is most prominently linked to asthma with reports on its expression and involvement in the development and repair of lung tissue (38, 39, 98). Periostin is indeed one of the most highly expressed genes in asthma (40).

Bronchial asthma is heterogeneous and can be classified into phenotypes based upon observable clinical or biological characteristics. The different phenotypes are now being defined as asthma endotypes. Various asthma endotypes fall into Th2^hi^ and Th2^lo^ clusters on the basis of the presence or absence of IL-4, IL-5, IL-13 and eosinophils in blood and tissues, respectively (40, 99). Periostin is a distinct signature protein for Th2^hi^ asthma (100). Most children and roughly 50% of adults have allergic asthma of Th2^hi^ endotype. The identification of the specific subtype is fundamental for optimizing the clinical intervention.

The biology of the Th2 response is a critical step towards the understanding of how T2 inflammation leads to disease pathology. The airway epithelial cells of Th2^hi^ patients are characterized by increased expression of three genes known to be up-regulated following IL-13 stimulation; periostin is one of these genes (40). IL-13 and IL-4 can both stimulate the secretion of periostin from lung fibroblast (10) and IL-13 can also induce epithelial cell production of periostin. Periostin also induces TGF-β signalling, which can further promote ECM deposition and airway remodelling (35). Sub-epithelial periostin promotes adherence and possibly migration of eosinophils into the lung (44).

**Figure 3:**
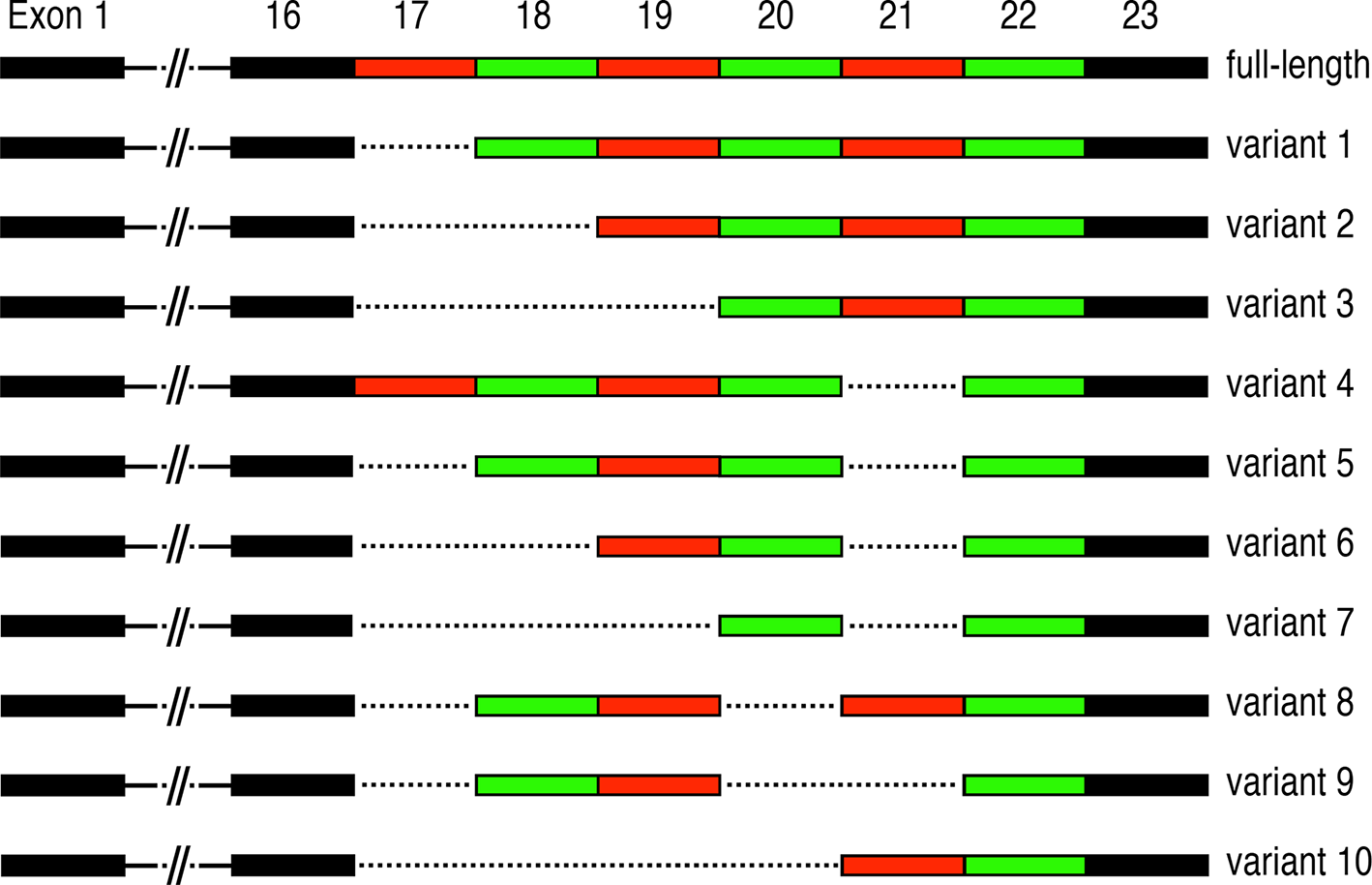
Periostin alternative splicing: Alternative splicing events occurring in the C-terminal region of periostin. Exons 17-22 are multiples of three nucleotides and are ranging from 90 (exons 18, 19), 84 (exon 21), 81 (exon 17), 78 (exon 20) to 42 (exon 22) nucleotides. Alternative splicing in this domain is likely to influence the binding abilities to different HSPGs and thus ECM dynamics and structure during inflammation, fibrosis and regeneration.

In asthma, airway remodelling is a common feature that results from the deposition of ECM and the proliferation of smooth muscle cells around large airways essentially narrowing the airway and constricting the airflow (101). Remodelling seems to be associated with severe and steroid-resistant asthma (102, 103), and includes epithelial thickening, myofibroblast differentiation and proliferation, smooth muscle thickening, goblet cell hyperplasia, changes in ECM composition and angiogenesis (102, 104). Periostin has been shown to be involved in at least three of these processes: myofibroblast differentiation and proliferation, changes in ECM composition and angiogenesis (41, 66, 79). In patients with asthma and pulmonary fibrosis, periostin has been localized in the basement membrane of the airway walls (10, 46). As mentioned above, IL-4 and IL-13 are responsible for the induction of periostin expression in bronchial asthma, with predominant localization in the thickened basement membrane in lungs of asthmatic patients, where periostin is thought to contribute to the development of sub-epithelial fibrosis (10). In this report, the authors also characterized periostin transcripts and found mainly three variants (see **Figure 3**). The main variant was the type containing the C-terminal domain exons 19 and 20 and lacking exons 17, 18, and 21 (variant 6, **Figure 3**). The second most abundant transcript additionally lacks exon 19 (variant 7), while the third most frequent transcript only lacks exons 17 and 21 (variant 5) (10). There is no statement regarding the potential functional implications of the expressed variants in this study. Interestingly, there are some controversial results from experiments in mice regarding the role of periostin in asthma. While some studies show that periostin plays a protective role in allergic respiratory diseases (105), one study using periostin homozygous mutant mice seems to implicate a reduction in airway allergic reactions in the absence of periostin (106). These controversial results have not been clarified so far, however they might be due to the use of different allergens, experimental setups and read-outs between the two studies.

## Chronic Rhinosinusitis (CRS)

CRS is a multifunctional upper respiratory disease that involves chronic inflammation of the nasal cavities and sinuses with a characteristic pattern of cytokine production and remodelling of inflamed tissue (107). CRS is divided into three subgroups based on clinical classification: CRS without nasal polyps, CRS with nasal polyps and allergic fungal rhinosinusitis (107). High levels of periostin expression were first described in sinonasal tissues of CRS patients by Stankovic et al. and later confirmed by other authors (55). In recent studies, periostin seemed to be associated more with eosinophilic CRS with nasal polyps (56), in a generic CRS patient cohort it seemed to be associated with remodelling changes and tissue eosinophilia (57), and it was also described as most likely derived from mast cells (MCs) (58). A review on periostin in CRS cites all relevant articles, however none of these contain any information about the expressed splice variants in CRS (59).

## Atopic Dermatitis (AD)

AD is a chronic relapsing, highly pruritic inflammatory skin disease (108, 109), characterized by epidermal hyperplasia, epithelial barrier dysfunction, infiltration of inflammatory cells (such as lymphocytes, eosinophils, and MCs) as well as fibrosis (110). Excessive Th2 cell responses to environmental stimuli are observed in the skin, particularly in the acute lesions. The pathogenesis of allergic skin inflammation in AD has been well characterized using various mouse models, for example Nc/Nga mice, RelB or Foxp3 deficient mice, thymic stromal lymphopoietin (TSLP) overexpressing mice or epicutaneous sensitization with HDM (for more details see *References*) (108). Using some of these models it was shown that periostin plays a crucial role in the onset of type 2 inflammation in an AD mouse model (37, 50). While IL-4 and IL-13 stimulate fibroblasts to produce periostin, periostin in turn directly stimulates the production of TSLP from keratinocytes (50). In AD, activated keratinocytes produce various pro-inflammatory cytokines and chemokines, important for the initiation or amplification of Th2 responses (111, 112) and periostin contributes to this *via* α_v_ integrin signalling, further fuelling the inflammation and disease progression. The IL-13/periostin/IL-24 pathway causes epithelial barrier dysfunction in AD (92). Furthermore, in an AD mouse model, IL-24 produced by keratinocytes is stimulated by IL-13 in a periostin-dependent manner *via* STAT-6 (92). In the current literature there is no information about periostin splice variants in relation to AD.

Interestingly, even though periostin is a known downstream molecule of IL-4 and IL-13, key cytokines of type 2 immune responses, and up regulation in and contribution to AD is established (see above) in a study on chronic spontaneous urticaria (CSU) periostin levels were found to be reduced in patients. The authors speculate that this unexpected finding may be due to the antihistamine treatment in CSU patients in their study (64), as histamine is known to directly induce periostin expression (113).

## Allergic Conjunctivitis (AC)

AC, also called ocular allergy, is a common allergic disorder and present in 30-71% of patients with allergic rhinitis (114). Upregulation of periostin and eosinophilic infiltration was observed in a mouse model of induced experimental AC, whereas the infiltration of eosinophils was diminished in periostin-deficient mice (53). Furthermore, periostin expression was significantly higher in patients with atopic keratoconjunctivits, vernal keratoconjunctivitis and seasonal AC compared to healthy donors. Periostin levels in tears were positively correlated with complications of atopic keratoconjunctivitis (52). While there is no information on the expressed periostin variants in relation to AC, investigations on the use of periostin as a tear biomarker for this condition are currently ongoing and appear very promising (54).

## Eosinophil-Associated Gastrointestinal Disorders (EGID) Including Eosinophilic Esophagitis (EoE)

EGID are characterized by an inappropriate accumulation of eosinophils within the gastrointestinal tract. It has been highlighted that Th2-driven cytokines play a role in EGID and children and adults with EGID often have positive skin testing for food allergens (115). The most common form of EGID is known as EoE, which is characterized by an infiltration of eosinophils within the esophagus (116). Interestingly, serum levels of periostin were not significantly elevated in patients compared to controls, however there was a correlation between elevated serum levels of IL-13 in conjunction with elevated periostin levels in a sup-population of patients (32). Animal models of EoE have demonstrated that IL-13 can induce EoE through a STAT-6-dependent mechanism (117). IL-13 appears to have an important role in the development of gastrointestinal eosinophilia as well as in the development of esophageal remodelling. Recently it was shown that α_M_β_2_ integrins mediate eosinophil tissue residency *via* periostin in EoE (33). There is no information on expressed splice variants of periostin in either EoE or EGID.

## Inflammatory Bowel Disease

Ulcerative colitis (UC) and Crohn’s disease (CD) are examples of IBD in which chronic inflammation of the digestive tract leads to persistent diarrhoea and increases the risk of tumour development in the intestine. UC and CD differ by the location and extent of inflammation, and periostin has been reported to be upregulated in UC and CD patients (29). In an induced IBD model, periostin deficient mice show a lower degree of inflammation compared to wild type mice (29). The precise functions of periostin in the development of IBD have so far not been elucidated. In a recent study, all expressed periostin isoforms in the small intestine were described. Interestingly, periostin variants 1, 5, 6 and 7 were detected but not the full length (see details in **Figure 3**) (28). Periostin has also been linked to the increased risk of colitis-associated colorectal cancer in a mouse model and its expression is also elevated in IBD patients (30). Epidemiologic studies highlight the increased risk of colorectal cancer (CRC) in IBD and generally link this risk to the pro-neoplastic effects of chronic intestinal inflammation (118). Given the altered expression of periostin in acute IBD and during remission in human patients (28), and the link to cancer progression in general as well as in CRC in mouse models, it is tempting to speculate that periostin may likely play a similar role in CRC in human patients (68, 74, 81).

One IBD complication is the development of conjunctivitis and other ocular problems in 4 to 10% of the patients, occurring more often in CD than UC (119). In view of the involvement of periostin in AC and in IBD, it would be interesting to investigate periostin expression levels in intestinal tissues and tears of IBD patients with and without ocular problems.

## Rheumatoid Arthritis (RA) and Ankylosing Spondylitis (AS)

RA is a chronic inflammatory disease affecting the joints caused by autoantibodies. Periostin has been shown to be upregulated in cells of the synovium and the synovial fluid in RA (17, 18). In cultured human chondrocytes, periostin induces the expression of IL-6, IL-8, MMP-1, MMP-3 and MMP13, which could be linked to the development of the disease (19). However, results presented at the ACR/ARHO meeting in 2017 by Yun-Hong Cheon, suggest that periostin has a protective function in RA. In three mouse models of artificially induced arthritis, periostin-deficient mice show a higher level of inflammation (20). These findings are in contradiction to the role periostin plays in other inflammatory diseases and further investigations are required to elucidate the role of periostin in RA. The studies relating to RA lack information about the presence of periostin splice variants.

AS is a form of arthritis that primarily affects the spine and sacroiliac (SI) joints, although other joints can become involved as well (120). Interestingly, an independent negative association between periostin and sclerostin level in AS patients was found, in line with murine studies of mechanical loading in which periostin up-regulates osteoblast activity through inhibition of sclerostin expression (21–23). Since periostin is produced by osteoblasts and is related to the production of new bone, it could be central to the process of excessive bone formation in active AS by inhibiting sclerostin. There is also a clear positive association of periostin with inflammatory markers and disease activity, i.e., higher periostin level with higher disease activity and higher systemic inflammation, but lower level with more extensive radiographic damage (21). Once again, all studies relating to these tissues lack a detailed analysis of periostin splice variants.

## Periostin Serum Level as a Diagnostic Marker

Many inflammatory diseases have a complex phenotype and are very heterogeneous, it is therefore important to find biomarkers for a precise medical approach to these diseases. Periostin expression has been linked to Th2 immunity and immune disorders. Because vascular structures run through the mesenchyme, proteins secreted basolaterally are excellent candidates for blood-based biomarkers of epithelial type-2 activation. Periostin is characterized as secreted from inflamed sites into various body fluids such as blood (121, 122), urine (123, 124) and tears (52), and a rise in periostin serum concentration reflects the increase in the inflamed sites (98).

Compared to urine and tears that are locally produced, serum has to be seen in a broader picture. Given that periostin is expressed by a large variety of cells and tissues at any given time one will be able to find it in the serum, generating a certain background level. In addition, periostin levels are age dependent and vary significantly during childhood and adolescence, to reach stable lower levels in the adulthood, thus influencing its usefulness as a marker during these developmental stages (125). In a recent study, an inverse association between circulating periostin and disease activity index in pediatric Crohn’s disease has been found, suggesting a more prominent role in repair rather than fuelling inflammatory cascades in this disease. The precise mechanism behind the significantly increased plasma levels of the protein during remission compared to active disease remains unclear (28). Periostin levels have been shown to be elevated in serum of asthmatic patients. However, not all asthmatic patients show this trend. It is widely accepted that asthma is not one single disease, rather a syndrome with different causes and features. The presence of periostin in serum is elevated in late-onset asthmatic patients compared to controls (121, 126), while no significant difference was observed in children. A possible explanation might be given by the already elevated level of periostin in the serum of children and teenagers compared to adults, most likely due to enhanced bone metabolism.

Elevated periostin serum levels reflect the Th2^hi^ eosinophilic inflammation in bronchial asthma. The identification of the specific asthma subtype is fundamental for optimizing the clinical intervention. Serum periostin is a distinct signature protein induced by IL-13 and has been utilized as a biomarker for the presence of a Th2^hi^ phenotype, which might predict responsiveness to IL-13 antagonism. In asthma, the efficacy of IL-13 antagonists was largely confined to the cohort presenting the IL-13^hi^ biomarker periostin (127). The serum level of periostin is a good indicator for the efficiency of the anti-IL-13 antibody lebrikizumab in the treatment of steroid-resistant asthma but may not be the ideal predictor for the Th2^hi^ phenotype (128).

Serum periostin is also increased in CRS, AD and AC (52, 129, 130). RA also showed elevated periostin levels but it has to be noted that the patients in this study were in remission, not acute cases (16).

Increased periostin levels have also been found in IPF and shown to be a good indicator for progression of the disease (41, 131). However, in a case of acute exacerbation of familial IPF there was no increase of periostin serum level, while high expression of periostin was detected in the fibrotic lesions in the lung (47). There are other allergic conditions, such as allergic rhinitis, in which no elevation of periostin level in the serum could be found (132).

These data show that periostin seems to have different functions in different allergic diseases and more refined experiments have to be conducted in order to fully understand these functions. A limitation in all these studies resides in the determination of the general serum levels of periostin. One way forward could consist in the analysis of the different isoforms, whether there are predominant isoforms in different allergic diseases and whether these isoforms can be found in the serum. It might well be that certain isoforms could be significantly elevated in the serum, while the overall periostin levels appear stable.

## Outlook

Matricellular proteins play an essential role in the regulation of the ECM and several cellular processes *via* matrix-cell-interaction. Genes encoding matricellular proteins, including periostin, have been shown to have on average four splice variations that can be translated into functional proteins, double the number compared to other genes in the genome (133). Noteworthily, there is growing evidence that splice variants of matricellular proteins play an important role in a variety of diseases (134–137).

Periostin exons 17 to 22 are made up of multiples of three nucleotides, thereby making it feasible for them to be spliced out without changing the reading frame and to be translated into protein. These isoforms only differ at their C-terminus, which has been implied to contain a nuclear localisation signal and to mediate interaction with HSPGs (3, 7). It has been shown that stimulation with neuregulin, a ligand for the erbB-receptor-family, can change the localisation of periostin in the cell (11), and that TGF-β can change the isoforms present in a cell (2). Given the strong interaction between TGF-β and periostin (138, 139), this indicates that different isoforms are located to different areas in the cell or the ECM. Up to now 11 different isoforms have been isolated from different tissues and different stages of development (see **Figure 3**) (1, 11, 140–143). Different isoforms have been shown to be important in different circumstances, for instance isoforms lacking exon 17 and 21 have been shown to be important in early stages of myocardial infarction (60). Certain isoforms have been linked to renal and non-small cell lung carcinoma (141, 143), while two isoforms, which are missing either exon 17 or 21, were found to promote angiogenesis (144). These data show that periostin isoform expression varies significantly between different tissues and different conditions, making the isoforms ideal targets for new, more specific treatment options.

Periostin has been linked to many allergic and inflammatory diseases, showing its importance in these conditions. In some cases, however, periostin is one of the causes or drivers of the disease (i.e., IBD or asthma), while in others periostin expression seems to be influenced by a primary infection exacerbating inflammation. There are also examples such as RA, in which periostin seems to have a protective function against inflammation. Therefore, further investigations are needed to analyse the precise role(s) of periostin in the different allergic and inflammatory diseases. In this respect, a promising direction could be the focus on the different isoforms of periostin. So far, although eleven isoforms have been found, no link to specific allergies or chronic inflammation diseases has been reported. Elucidating a correlation between specific periostin isoforms and inflammatory conditions could prompt towards new treatment options. This is very important, as treatment options are limited for several allergic and inflammatory diseases. In IBD for example, treatment options are aminosalicylate, immunosuppressants, antibiotics and specific antibody treatments. Anti-TNF antibodies are the most prominent treatment of IBD, however, they are only effective in about 50% of the patients and responsiveness to the treatment fades with time, reflecting the essential need for new specific drugs. Antibodies specific for periostin isoforms or compounds blocking specific interactions could represent possible alternatives in future treatments.

Currently available data reviewed here (see also **Table 1**) show that periostin levels in the blood are elevated in some diseases (i.e., asthma, CRS, AD and AC), while no change can be seen in other diseases such as familial IPF and allergic rhinitis, or an inverse correlation with disease activity was described as in pediatric Crohn’s disease. It would be interesting to analyse the isoform expression in all these conditions, possibly link specific isoforms to specific conditions and test if those isoforms are also elevated in the plasma. This would provide further insight to the function of the different isoforms and possibly help to further establish periostin levels in the blood as diagnostic markers for specific diseases.

## Author Contributions

All three authors provided input to the writing of this review by reading and analysing primary publications, on the content of the review as well as writing and editing of the review. DR edited and formatted the final version and created all figures. ES-R and DR generated the table. All authors contributed to the article and approved the submitted version.

## Funding

This review was written at the Nazarbayev University and the University of Aarhus. The review was supported by Nazarbayev University Faculty Development Grants (FDCRGP) to DR (090118FD5310), (021220FD2751) and to ESR (110119FD4510).

## Conflict of Interest

The authors declare that the research was conducted in the absence of any commercial or financial relationships that could be construed as a potential conflict of interest.

## Publisher’s Note

All claims expressed in this article are solely those of the authors and do not necessarily represent those of their affiliated organizations, or those of the publisher, the editors and the reviewers. Any product that may be evaluated in this article, or claim that may be made by its manufacturer, is not guaranteed or endorsed by the publisher.

## Abbreviations

AC, Allergic conjunctivitis; AD, Atopic dermatitis; AS, Ankylosing Spondylitis; CD, Crohn’s disease; CRS, Chronic Rhinosinusitis; CSU, Chronic spontaneous urticarial; ECM, extracellular matrix; EGID, Eosinophilic gastrointestinal disorder; EMI-domain, cysteine-rich domain; EMT, epithelial-mesenchymal transition; EoE, eosinophilic esophagitis; HSPGs, heparan sulfate proteoglycans; IBD, inflammatory bowel disease; IF, idiopathic pulmonary fibrosis; IL, interleukin; MMP, matrix metalloproteinases; UC, ulcerative colitis.
